# Dietary Macronutrients Do Not Differently Influence Postprandial Serum and Plasma Brain-Derived Neurotrophic Factor Concentrations: A Randomized, Double-Blind, Controlled Cross-Over Trial

**DOI:** 10.3389/fnins.2021.774915

**Published:** 2021-12-21

**Authors:** Elske Gravesteijn, Ronald P. Mensink, Ellen T. H. C. Smeets, Jogchum Plat

**Affiliations:** Department of Nutrition and Movement Sciences, NUTRIM School of Nutrition and Translational Research in Metabolism, Maastricht University Medical Center+, Maastricht, Netherlands

**Keywords:** brain-derived neurotrophic factor (BDNF), macronutrients, postprandial, serum, plasma, metabolism, human intervention

## Abstract

**Objectives:** Brain-derived neurotrophic factor (BDNF) plays a role in cognition and metabolism. Specific nutrients can affect fasting BDNF concentrations, which are potentially mediated by insulin and/or glucose. Since macronutrients trigger each a different insulin and glucose response, we examined postprandial effects of meals rich in fat, carbohydrates, or protein on BDNF concentrations. BDNF was analyzed in serum and plasma, since concentration differences can be found between matrices.

**Methods:** Healthy overweight/obese male participants (*n* = 18) participated in this randomized, double-blind, cross-over trial consisting of three test days with 1 week wash-out periods. Either a high-fat (En% fat, carbohydrates, protein: 52.3, 39.2, 8.0), high-carbohydrate (En% 9.6, 81.5, 8.6) or high-protein meal (En% 10.6, 51.5, 36.9) was consumed on each test day. BDNF concentrations were measured after 0, 60, and 240 min. Glucose and insulin concentrations were measured after 0, 15, 30, 45, 60, 90, 120, and 240 min.

**Results:** BDNF concentrations were higher in serum compared with plasma (*P* < 0.001). Postprandial BDNF concentrations in serum decreased significantly after the high-fat (*P* = 0.013) and high-carbohydrate meals (*P* = 0.040), and showed a trend after the high-protein meal (*P* = 0.076). No differences were found between meals (*P* = 0.66). Postprandial BDNF concentrations measured in plasma did not significantly change after the different meals (*P* = 0.47). As total area under the curve (AUC) for glucose was significantly higher after the high-carbohydrate meal compared with the high-fat (*P* = 0.003) and high-protein meals (*P* < 0.001), and the total AUC for insulin was higher after the high-carbohydrate (*P* < 0.001) and high-protein meals (*P* < 0.001) compared with the high-fat meal, it seems that acute changes in glucose and insulin do not affect postprandial BDNF concentrations. However, after the high-protein meal, the higher total AUC for glucose correlated with lower serum BDNF concentrations, and a higher maximal increase in glucose correlated with a lower maximal increase in plasma BDNF concentrations. There were no correlations with insulin concentrations after either meal.

**Conclusion:** Serum BDNF concentrations were higher than plasma concentrations. Since postprandial BDNF responses were not different between the meals, we conclude that there is no role for insulin or glucose in regulating postprandial BDNF concentrations.

**Clinical Trial Registration:** [www.ClinicalTrials.gov], identifier [NCT03139890].

## Introduction

Brain-derived neurotrophic factor (BDNF) is a protein that contributes to brain development in childhood, and positively affects neuronal functioning and cognition later in life (McAllister et al., 1999). The relevance of understanding the physiology of BDNF becomes even more relevant as it has been discovered that BDNF also plays a role in metabolism (Marosi and Mattson, 2014). Overall, higher circulating BDNF concentrations are associated with improved health (Miranda et al., 2019).

We have recently concluded in a systematic literature review that specific dietary components can influence circulating fasting BDNF concentrations (Gravesteijn et al., 2021). Amongst others, we concluded that a link could be possible between dietary macronutrient composition and fasting BDNF concentrations, which is potentially mediated by insulin. Moreover, Lee et al. (2018, 2020) showed a decrease in serum BDNF concentrations in older participants after an oral glucose tolerance test (OGTT). In addition, diabetic patients are characterized by lower BDNF concentrations as compared to healthy controls (Fujinami et al., 2008; Li et al., 2016). In other words, there is an apparent link between insulin and/or glucose concentrations and BDNF concentrations. So far, it remains unanswered whether BDNF relates to plasma insulin or plasma glucose concentrations, or both.

Next to dietary interventions evaluating changes in fasting BDNF concentrations, a number of studies also evaluated postprandial changes in BDNF concentrations. This can be of interest since it provides the possibility to differentiate between the role of insulin and glucose in BDNF changes, as insulin and glucose responses are different for each macronutrient: carbohydrates elevate both insulin and glucose, proteins also trigger the release of insulin but do not elevate glucose as pronounced as carbohydrates, and fats do not give a clear insulin or glucose response, but mostly elevate triacylglycerol (TAG) concentrations (Nuttall and Gannon, 1991). In this context, high protein diets induced BDNF expression in C57Bl/6 mice brain (Zeeni et al., 2018) and increased plasma BDNF levels in Wistar rats (Shafie et al., 2020). However, the effect of protein intake on circulating BDNF concentrations in humans has not been evaluated. In contrast to protein, effects of a high-fat diet have already been examined in humans. More specific, a high fat intake showed a decrease in plasma BDNF concentrations in healthy young male adults (Karczewska-Kupczewska et al., 2012). Finally, the earlier mentioned OGTT studies by Lee et al. (2018, 2020) showed a decrease in BDNF. Altogether, there are clear indications for differences in postprandial BDNF responses after intake of the three macronutrients, but designs, populations, and details of the meals in the different studies are too distinct to draw conclusions.

Therefore, the aim of this study was to investigate the influence of three different meals high in either one of the three macronutrients fat, carbohydrates, and protein on circulating postprandial BDNF concentrations in a systematic, side by side manner. Since peripheral BDNF is mainly stored in platelets (Eyileten et al., 2017), BDNF concentrations in the circulation highly vary depending on the matrix in which it is analyzed (Gravesteijn et al., 2021). It is, therefore, interesting to evaluate these postprandial changes after the three meals both in serum and plasma.

## Materials and Methods

### Participants

For this study, 20 apparently healthy overweight and obese male participants were recruited for their increased glucose and insulin postprandial responses from Maastricht and surrounding area. Inclusion criteria to evaluate eligibility for participation were: male gender, aged 18–70 years, BMI 25–35 kg/m^2^, stable body weight defined as weight gain or loss <3 kg in the past 3 months, non-smoker or smoking cessation >1 year, no medication targeting blood pressure, lipid or glucose metabolism, no diabetic patient, and no active cardiovascular disease. All participants gave their written consent before the start of the study. Study procedures were in accordance with the Declaration of Helsinki, and approved by the Medical Ethical Committee of the University Hospital Maastricht/Maastricht University (METC173012). The study was registered in May 2017 at ClinicalTrials.gov (NCT03139890).

### Study Design

The study had a randomized, double-blind, controlled cross-over design. In total, 20 participants participated in three postprandial test days, each separated by a minimum of 1-week wash-out period. The order of the three test meals was equally randomly assigned to participants by an independent researcher. Participants were instructed to refrain from strenuous physical activity 48 h before each test day, and from alcohol consumption 24 h before each test day. In addition, they were asked to keep their habitual food intake and physical activity levels constant during the entire study period.

### Postprandial Test Day

Participants arrived in the morning of the test days after an overnight fast (from 8 PM) to the Metabolic Research Unit Maastricht. To standardize the measurements, men were instructed to travel either by car or public transport, but identical on all three occasions. Upon arrival, first, an intravenous cannula was placed and a fasting blood sample (T0) was taken. Subsequently, participants consumed either the high-fat, high-carbohydrate or high-protein mixed meal. The meals had to be consumed in three stages: 1 min was provided to consume 1/3rd of the meal, which was followed by a 2-min break. This was repeated until the meal was consumed completely. When participants did not manage to consume the meal according to this protocol, the breaks were shortened to provide participants more time for the consumption periods.

The mixed meals were prepared on the morning of the test days by an independent researcher. All three meals contained 953 kilocalories each. The composition of the high-fat meal comprised of 52.3 En% from fat, 39.2 En% from carbohydrates, and 8.0 En% from protein, the high-carbohydrate meal of 9.6 En%, 81.5 En%, and 8.6 En%, respectively, and the high-protein meal of 10.6 En%, 51.5 En%, and 36.9 En%. The meal compositions are shown in Table 1. Depending on the meal’s volume participants received a certain amount of water separately to assure that the total volume of each meal was 730 mL. Subsequent postprandial blood samples were collected at the following time points after meal consumption: 15 min (T15), 30 min (T30), 45 min (T45), 60 min (T60), 90 min (T90), 120 min (T120), and 240 min (T240).

**TABLE 1:** Meal composition of the high-fat, high-carbohydrate, and high-protein meals.

Nutritional value	High-fat meal	High-carbohydrate meal	High-protein meal
Energy (kcal)	953	953	953
Cholesterol (mg)	331.2	334.1	334.1
Fat (g)	55.4	10.2	11.3
- SFA (g)	33.1	3.4	4.0
- MUFA (g)	16.0	4.0	4.1
- PUFA (g)	5.0	0.9	0.9
**Total fat (En%)**	**52.3**	**9.6**	**10.6**
Carbohydrates (g)	93.5	194.3	122.7
- Glucose (g)	0.3	0.3	1.2
- Sucrose (g)	45.6	145.6	59.1
- Lactose (g)	0.5	0.0	1.3
- Polysaccharide (g)	45.0	45.0	58.1
**Total carbohydrates (En%)**	**39.2**	**81.5**	**51.5**
Protein (g)	19.2	20.4	87.9
**Total protein (En%)**	**8.0**	**8.6**	**36.9**
**Total volume (g)**	**433**	**468**	**615**
Water (mL)	297	262	115

*SFA, saturated fatty acids; MUFA, monounsaturated fatty acids; PUFA, polyunsaturated fatty acids. The bold values are the totals.*

### Biochemical Analyses

Blood was collected in serum STT-II advance tubes (Becton Dickinson) for the analyses of TAG (GPO Trinder, Sigma-Aldrich Corp., St. Louis, MO, United States) at T0, T30, T60, T120, T180, and T240, with correction for free glycerol, and insulin at all time points (human insulin specific RIA kit, Millipore, Billerica, MA, United States). The minimum clotting time was 30 min at room temperature. To obtain serum, the tubes were centrifuged at 1,300 × *g* for 15 min at 21°C. In addition, blood was collected in EDTA, heparin, and NaF tubes. NaF samples were used for the analyses of glucose (Glucose HK CP, Horiba ABX, Montpellier, France), and free fatty acids (FFA; NEFA-HR, FUJIFILM Wako Diagnostics U.S.A. Corp., Mountain View, CA, United States) at all time points. To obtain NaF plasma, the tubes were immediately centrifuged at 1,300 × *g* for 15 min at 4°C. Finally, EDTA and heparin tubes were processed based on the same protocol as the NaF tubes. After centrifugation, all serum and plasma samples were portioned into aliquots, snap-frozen in liquid nitrogen, and stored at −80°C until biochemical analyses after the study was completed. Both serum and plasma (EDTA and heparin) samples were used for the analyses of BDNF at T0, T60, and T240 by an enzyme-linked immunosorbent assay (Duo Kit ELISA, R&D Systems, Minneapolis, MN, United States) according instructions by manufacturer. The ELISA cannot distinguish between precursor BDNF (proBDNF) and mature BDNF (mBDNF). Therefore, BDNF concentrations reported here are the sum of proBDNF and mBDNF.

### Statistical Analyses

Data are presented as means ± standard deviation (SD), unless indicated otherwise. Despite the research objective is not part of the primary outcome measure, the initial statistical plan was followed to evaluate the postprandial effects of the macronutrients fat, carbohydrates, or protein on BDNF concentrations and other biomarkers. Fasting values between the three test days were compared using repeated-measures ANOVA. In order to test the pattern of the dependent variable after each of the three meals over time, postprandial curves were analyzed with linear mixed models. For this, the absolute BDNF concentration was used as dependent variable with time as fixed factor. If statistically significant, time points T60 and T240 were compared to fasting values with Bonferroni adjustment for multiple comparisons. Next, to compare the patterns of BDNF after the three meals side-by-side, again a linear mixed model analysis was performed, now with the changes in BDNF concentrations as dependent variable and with period, time, diet, and interaction (time × diet) as fixed factors. When the interaction term was not statistically significant, it was excluded from the model. Next, total area under the curve (total AUC) was calculated for all parameters. Incremental area under the curve (iAUC) was calculated for glucose, TAG, insulin, and plasma BDNF. Decremental area under the curve (dAUC) was calculated for FFA, and serum BDNF. Maximal increases or decreases were calculated by comparing the changes between the time points relative to fasting values. In order to compare the AUC data between the three meals a Friedman test was used. Bonferroni corrections were applied to correct for multiple comparisons. Relations between the AUC data for BDNF and glucose, TAG, insulin, and FFA were analyzed with Spearman correlation coefficients. Results were considered statistically significant at *P* < 0.05. Statistical analyses were performed using SPSS (IBM Corp., IBM SPSS Statistics for Windows, version 26.0, Armonk, NY, United States).

## Results

### Study Participants

During the study, two participants dropped out due to personal reasons, which implies that a total of 18 men with a median (interquartile range) age of 65 (51–67) years and a BMI of 30.5 ± 2.9 kg/m^2^ completed the study. All baseline characteristics are presented in Supplementary Table 1.

### Postprandial Brain-Derived Neurotrophic Factor Concentrations

As shown in Table 2 and Supplementary Figure 1, fasting BDNF concentrations were not significantly different between the three test days, in both serum (*P* = 0.769) and EDTA plasma (*P* = 0.858) samples. Interestingly, fasting BDNF concentrations in serum samples were 4- to 5-fold higher as compared to fasting BDNF concentrations in EDTA plasma samples (*P* < 0.001). When visually inspecting the data, the overall pattern indicated that serum BDNF concentrations decreased till 240 min postprandial after all three meals, whereas plasma BDNF concentrations increased during the same postprandial interval after all three meals. Fasting BDNF concentrations in heparin plasma were even lower as compared to EDTA plasma (*P* < 0.001), but showed the same time pattern as compared to EDTA plasma (Supplementary Table 2 and Supplementary Figure 1C).

**TABLE 2:** BDNF concentrations before and after consumption of the high-fat, high-carbohydrate or high-protein meals in serum and plasma (EDTA).

Medium	Meal	BDNF (pg/ml)		
		T0	T60	T240
Serum	Fat*	24,290 ± 8,842	19,120 ± 7,723	16,366 ± 6,793^b^
	Carbohydrates*	24,073 ± 9,227	18,502 ± 8,733	16,535 ± 8,869^b^
	Protein^a^	22,559 ± 10,077	21,229 ± 8,164	16,199 ± 7,495
EDTA	Fat	5,154 ± 2,002	5,444 ± 2,440	5,977 ± 1,948
	Carbohydrates	5,072 ± 1,549	5,084 ± 1,879	5,833 ± 1,947
	Protein	5,335 ± 1,886	5,460 ± 1,931	5,256 ± 1,876

*Values are presented as means ± SD.*

*Significant time effect after the high-fat (P < 0.05) and high-carbohydrate meal (P < 0.05).

*^a^Trend after the high-protein meal (P = 0.076).*

*^b^Significant time effect at time point T240 compared to T0 (P < 0.05) after Bonferroni correction.*

**FIGURE 1:**
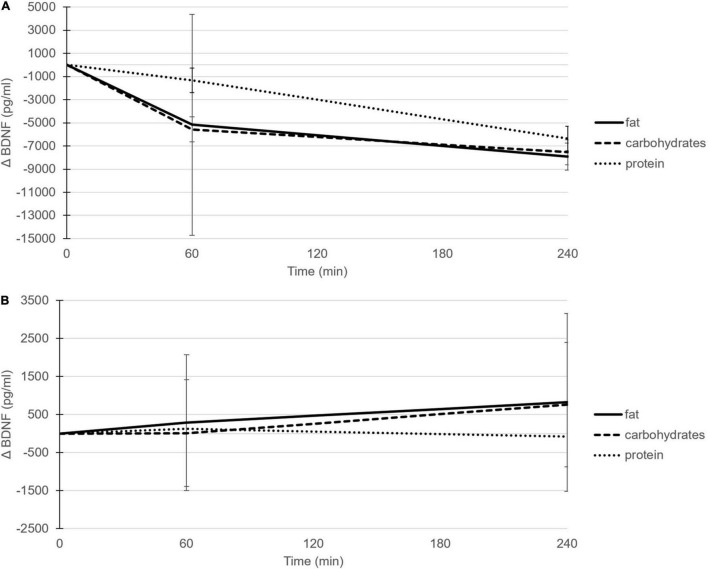
Postprandial mean changes (±SD) in BDNF concentrations (pg/ml) after consumption of the high-fat (solid line), high-carbohydrate (dashed line) and high-protein (dotted line) meal in serum **(A)** and EDTA plasma **(B)**.

Within the serum samples, the postprandial changes in BDNF concentrations showed a significant time effect after the high-fat meal (*P* = 0.013; Table 2, Figure 1A, and Supplementary Figure 1A) and the high-carbohydrate meal (*P* = 0.040), whereas the response after the high-protein meal showed a trend (*P* = 0.076). More specific, the time effects after the high-fat and high-carbohydrate meals were significant at time point T240 as compared to T0 (*P* = 0.008 and *P* = 0.029, respectively). Comparing the postprandial responses relative to fasting concentrations side-by-side indicated no significant difference between the three meals (*P* = 0.662).

In contrast to the patterns shown in serum, the postprandial changes in BDNF concentrations within EDTA plasma samples did not show a time effect after any of the three meals; after the high-fat meal (*P* = 0.509; Figure 1B and Supplementary Figure 1B), after the high-carbohydrate meal (*P* = 0.355), and after the high-protein meal (*P* = 0.949). Again, no significant differences in postprandial plasma BDNF concentrations were found between meals (*P* = 0.473). In line with EDTA plasma samples, also the postprandial changes in BDNF concentrations within heparin plasma did not show a time effect after the high-fat (*P* = 0.224), high-carbohydrate (*P* = 0.083), or high-protein meal (*P* = 0.909), and no significant differences were found between meals (*P* = 0.499).

When the postprandial concentrations and changes in BDNF concentrations in serum (Table 3) and EDTA plasma (Table 4) were used to calculate the total AUC, dAUC, iAUC, and maximal increases or decreases, it appeared that the total AUC was not significantly different between the three meals (*P* = 0.607 and *P* = 0.486, for serum and EDTA plasma, respectively). No significant differences were found for the dAUC in serum (*P* = 0.642) or the iAUC in EDTA plasma (*P* = 0.073), neither for the maximal decreases (*P* = 0.958) or the maximal increases (*P* = 0.146), respectively. These postprandial BDNF parameters in heparin plasma showed the same pattern as observed in EDTA plasma, but were even lower (Supplementary Table 3).

**TABLE 3:** Postprandial responses (dAUCs) and maximal decreases of BDNF concentrations (pg/ml) after consumption of the high-fat, high-carbohydrate or high-protein meal in serum.

Meal	Total AUC (concentration/240 min)	dAUC (concentration/240 min)	Max decrease
Fat	4,285,485 (3,369,833–5,667,180)	795,871 (109,779–2,888,708)	9,110 (2,817–19,988)
Carbohydrates	4,125,105 (3,166,148–5,197,710)	1,887,510 (146,304–2,683,538)	11,548 (2,860–17,534)
Protein	4,591,665 (4,168,493–5,487,653)	863,347 (125,501–1,656,218)	9,065 (4,456–14,669)

*Values are presented as median with ranges (25–75th percentiles).*

**TABLE 4:** Postprandial responses (iAUCs) and maximal increases of BDNF concentrations (pg/ml) after consumption of the high-fat, high-carbohydrate or high-protein meal in EDTA plasma.

Meal	Total AUC (concentration/240 min)	iAUC (concentration/240 min)	Max increase
Fat	1,296,285 (1,016,633–1,770,825)	142,899 (5,164–324,556)	1,092 (308–2,408)
Carbohydrates	1,380,855 (1,033,763–1,549,095)	87,512 (23,925–235,238)	1,037 (255–1,763)
Protein	1,238,220 (1,004,618–1,556,190)	38,363 (0–174,233)	439 (0–962)

*Values are presented as median with ranges (25–75th percentiles).*

### Postprandial Lipemia and Glycemia

Fasting glucose, TAG, and FFA concentrations at the start of the three test days were comparable, but fasting insulin concentrations were significantly higher at the start of the day of the high-protein meal compared to the high-fat meal (*P* = 0.003) and the high-carbohydrate meal (*P* = 0.046; Table 5 and Supplementary Figure 2). The total AUC based on the postprandial glucose concentrations was higher after the high-carbohydrate meal compared to the high-fat meal (*P* = 0.003) and the high-protein meal (*P* < 0.001). Consequently, also the iAUC and maximal increases in glucose concentrations were higher after the high-carbohydrate meal compared to the high-fat meal (*P* < 0.001 and *P* < 0.001, respectively) and the high-protein meal (*P* < 0.001 and *P* < 0.001, respectively). The total AUC based on the postprandial insulin concentrations was lower after the high-fat meal compared to the high-carbohydrate meal (*P* < 0.001) and the high-protein meal (*P* < 0.001), but no significant differences were found between the high-carbohydrate and high-protein meal (*P* = 0.134). Moreover, the iAUC and maximal increases for insulin were also lower after the high-fat meal compared to the high-carbohydrate meal (*P* < 0.001 and *P* < 0.001, respectively) and the high-protein meal (*P* = 0.002 and *P* = 0.001, respectively), but again no significant differences were found between the high-carbohydrate and high-protein meal (*P* = 0.096 and *P* = 0.739, respectively). The total AUC based on the postprandial TAG concentrations were not different between the meals (*P* = 0.311). However, the iAUC for TAG tended to be different between the meals (*P* = 0.092). The difference in maximal increases did reach significance between the high-fat and high-carbohydrate meal (*P* = 0.008). The total AUC based on the postprandial FFA concentrations tended to be different between the meals (*P* = 0.092). The dAUC and maximal decreases for FFA were lower after the high-fat meal compared to the high-carbohydrate meal (*P* < 0.001 and *P* < 0.001, respectively) and the high-protein meal (*P* = 0.003 and *P* = 0.005, respectively).

**TABLE 5:** Postprandial responses (iAUCs) and maximal increases of glucose, TAG, and insulin, and postprandial responses (dAUCs) and maximal decreases of FFA after consumption of the high-fat, high-carbohydrate or high-protein meal.

Blood marker	Meal	Fasting	Total AUC (concentration/240 min)	iAUC/dAUC (concentration/240 min)	Max increase/decrease
Glucose (mmol/L)	Fat	5.35 (5.11–5.73)	1,343 (1,231–1,422)	67 (43–108)	1.33 (1.14–1.89)
	Carbohydrates	5.53 (5.26–5.66)	1,428 (1,267–1,589)^b^	106 (59–263)^b^	2.31 (1.92–3.38)^b^
	Protein	5.42 (5.26–5.65)	1,328 (1,202–1,456)^c^	49 (19–97)^c^	1.17 (0.85–1.47)^c^
TAG (mmol/L)	Fat	1.16 (0.89–1.36)	382 (280–441)	82 (55–119)	0.76 (0.60–1.04)
	Carbohydrates	1.17 (0.76–1.87)	323 (226–565)	63 (38–108)	0.51 (0.26–0.92)^b^
	Protein	1.10 (0.78–1.51)	297 (244–453)	56 (32–114)	0.57 (0.30–1.01)
Insulin (μU/mg)	Fat	9.88 (7.90–0.57)^a^	7,767 (5,895–12,318)^a^	5,746 (4,126–9,376)^a^	59.06 (41.39–94.98)^a^
	Carbohydrates	10.52 (8.55–11.57)	12,905 (10,323–21,478)^b^	10,851 (7,808–18,549)^b^	106.79 (73.96–157.58)^b^
	Protein	11.11 (9.08–17.29)^c^	11,824 (8,594–17,281)	8692.43 (6198.06–14350.26)	98.70 (58.01–134.19)
FFA (μmol/L)	Fat	254.99 (147.66–328.30)	67,465 (32,598–93,810)	11,533 (4,432–2,7695)^a^	112.53 (51.09–188.87)^a^
	Carbohydrates	288.98 (161.38–371.62)	112,788 (52,729–140,852)	43,671 (17,951–54,575)^b^	237.67 (118.36–306.21)^b^
	Protein	260.07 (171.02–310.22)	85,939 (44,926–109,584)	26,574 (13,026–39,299)	189.20 (101.61–225.13)

*Values are presented as median with ranges (25–75th percentiles); TAG, triacylglycerol; FFA, free fatty acids.*

*^a^Significant difference between high-fat and high-protein meal.*

*^b^Significant difference between high-fat and high-carbohydrate meal.*

*^c^Significant difference between high-carbohydrate and high-protein meal.*

### Correlations Between Brain-Derived Neurotrophic Factor, Lipemia, and Glycemia

The total AUC for BDNF in serum samples correlated with the total AUC for the glucose response after the high-protein meal (r = 0.486; *P* = 0.041), which indicates that a higher total AUC for glucose is associated with lower BDNF responses. No correlations were found between the total AUC of BDNF in serum samples and TAG, insulin, and FFA after one of the meals. Moreover, there were no correlations between the dAUC or the maximal decrease of BDNF in serum and glucose, TAG, insulin, and FFA after one of the meals. For the EDTA plasma samples, a correlation was found between the total AUC of BDNF and the total AUC of FFA after the high-carbohydrate meal (*r* = −0.478; *P* = 0.045), which indicates that higher FFA responses are associated with lower BDNF responses. No correlations were found between the total AUC of BDNF in EDTA samples and glucose, TAG, and insulin after one of the meals. A correlation was found between the maximal increase of BDNF in plasma samples and glucose after the high-protein meal (*r* = −0.701; *P* = 0.001), which indicates that higher maximal increases of glucose are associated with lower maximal increases of BDNF. No correlations were found between the iAUC or the maximal increase of BDNF in plasma samples and glucose, TAG, insulin, and FFA after one of the meals.

## Discussion

Based on the literature, we hypothesized that postprandial BDNF responses after intake of fat, carbohydrate and protein rich meals were potentially different because of the suggested role for insulin and/or glucose in BDNF regulation. Variations in designs, populations and meal composition between those studies made it difficult to be conclusive. Therefore, we here evaluated the influence of the three macronutrients on postprandial BDNF concentrations side by side. We found that BDNF concentrations significantly decreased postprandially in serum, but not in plasma. However, no differences were found between the three meals rich in either one of the macronutrients, in serum or plasma. Since postprandial plasma insulin and glucose concentrations were clearly distinct between the three meals, as expected, this also implies that both insulin and glucose, at least in the acute situation, are not involved in regulating circulating BDNF concentrations. However, it should be considered that measuring postprandial BDNF was not the primary end point for which this study was initially designed and powered.

Although we hypothesized that insulin and/or glucose regulates circulating BDNF concentrations, there are also arguments to suggest the opposite, i.e., that BDNF regulates metabolic control, thereby affecting insulin sensitivity (Rozanska et al., 2020). In more detail, in obese diabetic C57BL/KsJ mice BDNF administration increased pancreatic beta cells sensitization (Yamanaka et al., 2006). Furthermore, in Zucker diabetic fatty rats BDNF administration lowered hepatic gluconeogenesis (Kuroda et al., 2003). Finally, de Luis et al. (2018) showed that specific BDNF gene variants are associated with insulin resistance in humans. Interestingly, plasma BDNF concentrations were negatively correlated with insulin resistance using the homeostatic model assessment version 2 (HOMA2-IR), but not with insulin concentrations (Krabbe et al., 2007). More mechanistically, BDNF and insulin are involved in overlapping signaling pathways *via* activation of protein kinase B (Foulstone et al., 1999). Therefore, we postulated that it could also be that insulin affects circulating BDNF concentrations instead of the other way around as others postulated. However, our data could not confirm this assumption, since changes in insulin concentrations were not aligned with changes in BDNF concentrations.

Instead of the potential direct effect of insulin, as suggested from the above-mentioned overlapping signaling pathways, such an effect of insulin on BDNF concentrations could also be indirect *via* changing postprandial plasma glucose or FFA concentrations. Notwithstanding the differences in postprandial glucose concentrations between meals, we were not able to show a difference in BDNF concentrations after the high-carbohydrate meal compared to the high-protein meal. However, there was a correlation between the total AUC for serum BDNF and the total AUC for glucose, and between the maximal increase of plasma BDNF and the maximal increase of glucose after the high-protein meal, indicating a possible indirect effect of insulin on BDNF concentrations *via* plasma glucose concentrations.

Besides the glucose axis, insulin could also have an indirect effect on BDNF concentrations *via* changes in plasma FFA concentrations. Indeed, we found a correlation between the total AUC for plasma BDNF and the total AUC for FFA after the high-carbohydrate meal. Insulin inhibits lipolysis, thereby decreasing FFA concentrations. Conversely, elevated FFA concentrations induce insulin resistance (Dresner et al., 1999). The results of a postprandial analysis by Karczewska-Kupczewska et al. (2012) showed indeed that an elevation of FFA by a high-fat meal decreased plasma BDNF concentrations in healthy young male adults. In this same study, a euglycemic hyperinsulinemic clamp with intralipid infusion decreased both insulin sensitivity and BDNF concentrations as compared to a clamp without intralipid infusion. However, a high-fat meal does not substantially affect insulin concentrations as a high-carbohydrate or high-protein meal do. This could mean that the changes in BDNF concentrations seen in the study by Karczewska-Kupczewska et al. (2012) could be attributed to the direct effect of FFA but not to the indirect effect of insulin *via* FFA.

Finally, it could also be possible that postprandial TAG concentrations affect BDNF concentrations. However, to the best of our knowledge, there is lack of evidence connecting postprandial changes in TAG and BDNF concentrations. Also in the current study, despite the increased TAG response after the high-fat meal no difference in changes in BDNF concentrations were found between the meals, which excludes a possible role for TAG.

Based on our findings presented here, it seems that there is no role for insulin and/or glucose in changing postprandial BDNF. Therefore, the question now is how to explain the earlier findings showing that insulin resistance or higher glucose levels in diabetic patients is associated with lower circulating BDNF concentrations in both serum (Fujinami et al., 2008; Li et al., 2016) and plasma (Krabbe et al., 2007). The most likely explanation is that the acute effects of increased postprandial insulin or glucose in this postprandial situation, are not strong enough to induce changes in BDNF concentrations, whereas long term exposure to chronically elevated glucose and/or insulin, as seen in diabetic patients, might have such an effect. However, it seems a bit more complex since prediabetes is associated with higher BDNF concentrations (Liu et al., 2016). Moreover, higher BDNF concentrations were also found in untreated newly diagnosed diabetic patients (Suwa et al., 2006), but as well as in previous diagnosed type 2 diabetic patients (Civelek et al., 2013; Boyuk et al., 2014). In the latter two studies, though, it should be considered that the control group had a lower weight which could indicate that BDNF concentrations were influenced by BMI (Li et al., 2016), albeit evidence is controversial. Finally, these apparent discrepancies could also relate to ethnic differences between study populations (Fujinami et al., 2008; Boyuk et al., 2014). Altogether, this at least indicates that the diabetic stage influences BDNF concentrations (Rozanska et al., 2020). However, it is difficult to conclude how this is regulated. Moreover, it also implies that in our study population of healthy overweight or obese participants, postprandial effects of insulin and glucose on BDNF might be compensated. After all, it is also still a possibility that the relation between insulin and BDNF is actually in the other direction, i.e., BDNF concentrations affecting insulin, as suggested from animal studies, which might not be found in healthy insulin sensitive participants.

Interestingly, both fasting and postprandial BDNF concentrations were higher in serum than plasma, which can be explained by the fact that BDNF is mainly stored in platelets and released during clotting (Rosenfeld et al., 1995). However, the interesting observation was that serum BDNF concentrations showed a significant postprandial decrease, whereas plasma BDNF concentrations did not show a significant postprandial change. Therefore, it could be that either there may be less platelets in the postprandial state or the clotting process, in which BDNF is released from platelets, is less efficient in the samples collected during the postprandial state. In this context, it was found that a high-fat meal elevated platelet count (Sinzinger and Berent, 2012), but a high-carbohydrate meal did not (Ahuja et al., 2009). Regarding the clotting process, it has been shown that meals rich in fats seem to activate coagulation factor VII which stimulates the clotting process (Silveira, 2001). Hyperglycemia also activates the coagulation process (Onbaşi et al., 2016), in contrast to meals rich in proteins (Silveira, 2001). In other words, when the postprandial clotting process is indeed activated more after the high-fat and high-carbohydrate meals, higher postprandial serum BDNF concentrations would have been expected as compared to the high-protein meal. Moreover, since postprandial insulin also stimulates platelet activation, platelet activation upon hyperglycemia seems to be actually triggered by hyperinsulinemia (Spectre et al., 2012). As proteins trigger the release of insulin, higher postprandial serum BDNF concentrations could be expected after the high-protein meal as well. Therefore, this does not seem to be a likely explanation for the observed decrease in serum BDNF concentrations after all three macronutrient rich meals. More important, however, if we assume that the clotting process in serum samples was complete, this means that the previously mentioned postprandial changes in clotting factors are unlikely to affect serum BDNF concentrations, but would rather have affected plasma BDNF concentrations.

## Conclusion

We have demonstrated that macronutrients do not differently affect BDNF concentrations in the postprandial state. We found that postprandial BDNF concentrations decreased and were higher in serum compared to plasma. Based on these differences, we postulate to analyze BDNF concentrations in future studies at least in serum, and preferable in both serum and plasma to enlarge our understanding of BDNF regulation. Although it seems there is no direct role for insulin or glucose on BDNF, at least not in the acute situation, it would be valuable that future studies focus on the role of insulin on BDNF in the long-term situation.

## Data Availability Statement

The raw data supporting the conclusions of this article will be made available by the authors, without undue reservation.

## Ethics Statement

The studies involving human participants were reviewed and approved by the Medical Ethical Committee of the University Hospital Maastricht/Maastricht University. The patients/participants provided their written informed consent to participate in this study.

## Author Contributions

All authors listed have made a substantial, direct, and intellectual contribution to the work, and approved it for publication.

## Conflict of Interest

The authors declare that the research was conducted in the absence of any commercial or financial relationships that could be construed as a potential conflict of interest.

## Publisher’s Note

All claims expressed in this article are solely those of the authors and do not necessarily represent those of their affiliated organizations, or those of the publisher, the editors and the reviewers. Any product that may be evaluated in this article, or claim that may be made by its manufacturer, is not guaranteed or endorsed by the publisher.

## Abbreviations

AUC: area under the curve
BDNF: brain-derived neurotrophic factor
dAUC: decremental area under the curve
FFA: free fatty acids
HOMA2-IR: homeostatic model assessment for insulin resistance version 2
iAUC: incremental area under the curve
OGTT: oral glucose tolerance test
SD: standard deviation
TAG: triacylglycerol.
