# Supplementary material for: Dietary Macronutrients Do Not Differently Influence Postprandial Serum and Plasma Brain-Derived Neurotrophic Factor Concentrations: A Randomized, Double-Blind, Controlled Cross-Over Trial
Source: Front Neurosci. 2021 Dec 21;15:774915. doi: 10.3389/fnins.2021.774915 (PMC8724042; doi:10.3389/fnins.2021.774915)
Supplement: Supplementary file 1 [file Data_Sheet_1.PDF]

**Supplemental Table 1**. Baseline participant characteristics

| | **Participants (n=18)** |
|---|---|
| Age (years) | 65 (51 – 67)[*] |
| BMI ($kg/m^2$) | 30.5 ± 2.9 |
| Fasting glucose (mmol/L) | 5.67 ± 0.49 |
| Fasting TAG (mmol/L) | 1.27 ± 0.47 |
| Fasting insulin (μU/mg) | 12.19 ± 6.13 |
| Fasting FFA (μmol/L) | 308.12 ± 133.21 |

*Values are presented as means ± SD; BMI: body mass index; TAG: triacylglycerol; FFA: free fatty acids; [*] median (interquartile range)*

**Supplemental Table 2**. BDNF concentrations before and after consumption of the high-fat, high-carbohydrate or high-protein meal in heparin plasma

| **Medium** | **Meal** | **BDNF (pg/ml)** | | |
|---|---|---|---|---|
| | | **T0** | **T60** | **T240** |
| **Heparin** | Fat | 828 ± 522 | 1093 ± 872 | 1307 ± 990 |
| | Carbohydrates | 862 ± 740 | 942 ± 413 | 1344 ± 813 |
| | Protein | 1139 ± 1032 | 1260 ± 1140 | 1274 ± 888 |

*Values are presented as means ± SD*

**Supplemental Table 3**. Postprandial responses (iAUCs) and maximal increases of BDNF concentrations (pg/ml) after consumption of the high-fat, high-carbohydrate or high-protein meal in heparin plasma

| Meal | Total AUC (concentration/240 min) | iAUC (concentration/240 min) | Max increase |
|---|---|---|---|
| **Fat** | 243555 (157088 – 368423) | 37087 (591 – 140250) | 271 (24 – 929) |
| **Carbohydrates** | 247065 (176288 – 313260) | 71880 (26480 – 153623) | 533 (176 – 1060) |
| **Protein** | 236445 (163193 – 324045) | 42360 (1417 – 71562) | 226 (26 – 464) |

*Values are presented as median with ranges (25-75th percentiles)*

**Supplemental Figure 1**. BDNF concentrations before and after consumption of the high-fat, high-carbohydrate or high-protein meal in serum (A), EDTA plasma (B), and heparin plasma (C)

**A**

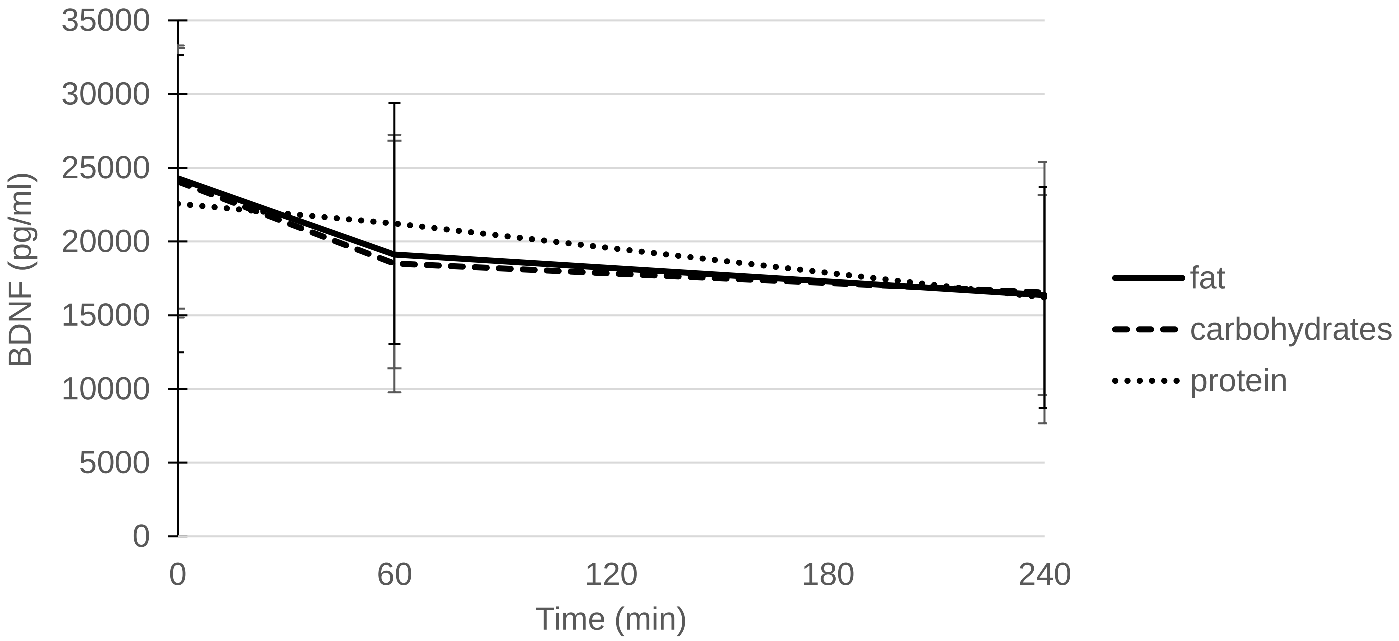


**B**

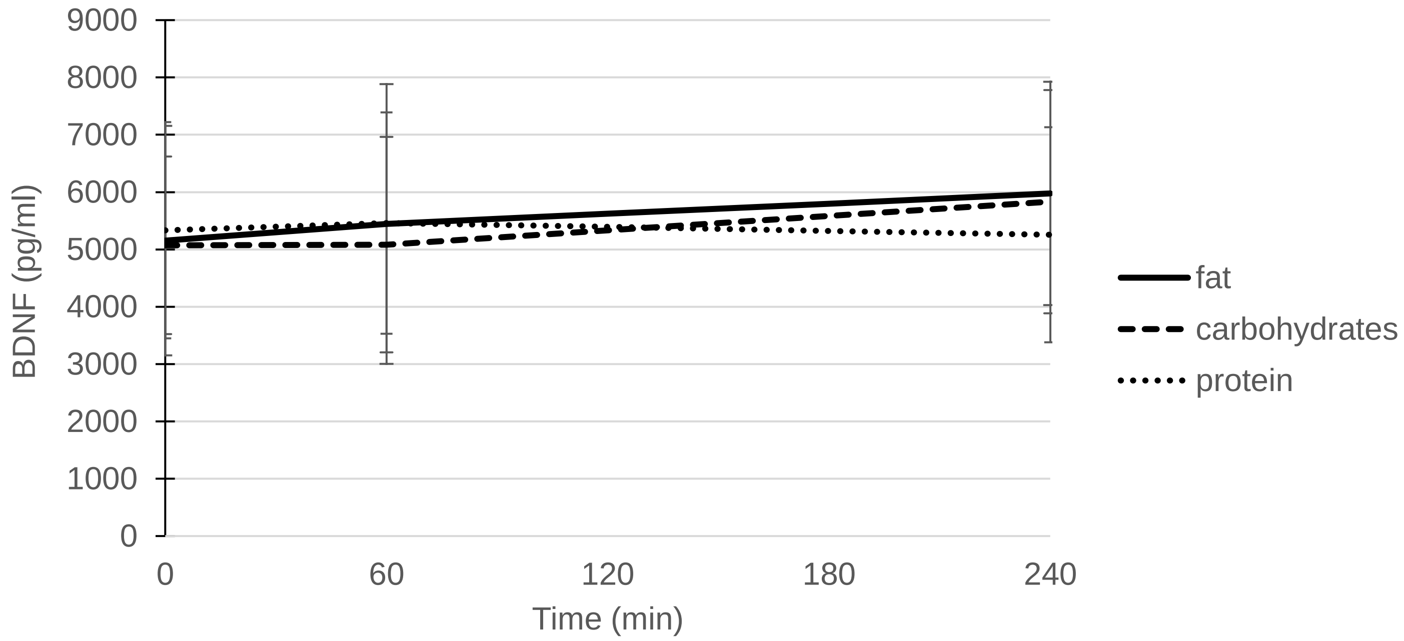

**C**

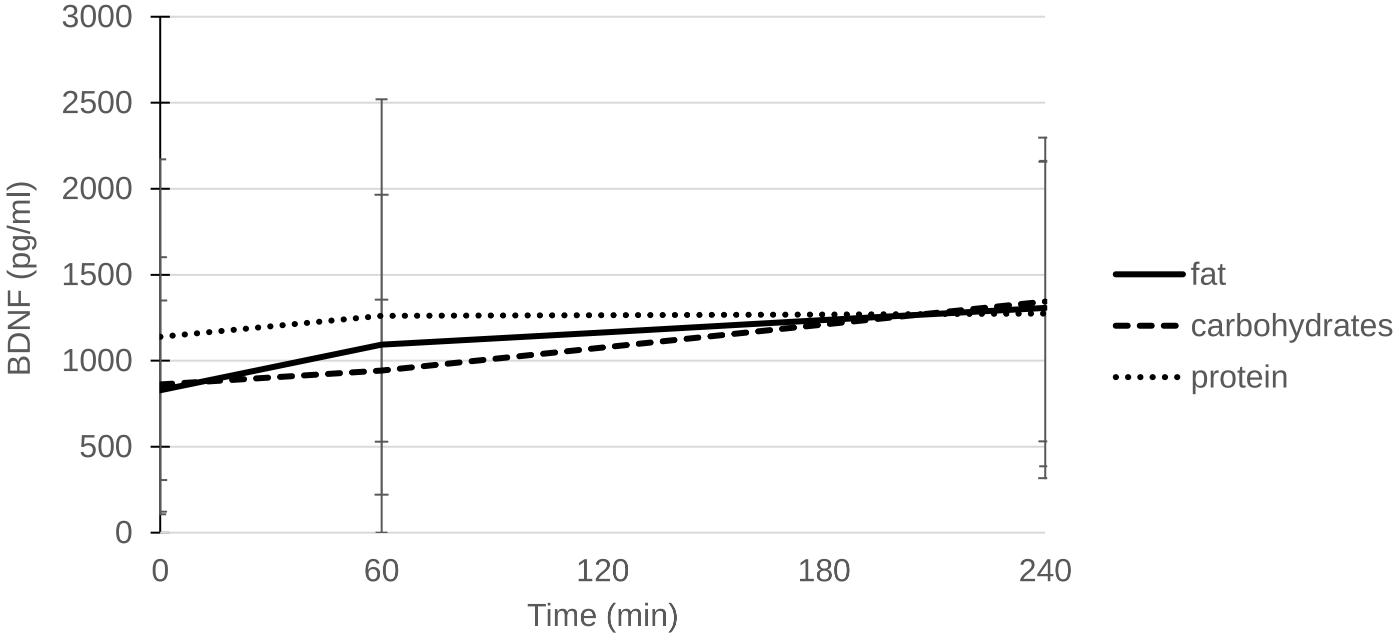

3000
2500
2000
1500
1000
500
0
BDNF (pg/ml)
0
60
120
180
240
Time (min)
fat
carbohydrates
protein

**Supplemental Figure 2**. Glucose (A), TAG (B), insulin (C), and FFA (D) concentrations before and after consumption of the high-fat, high-carbohydrate or high-protein meal

**A**

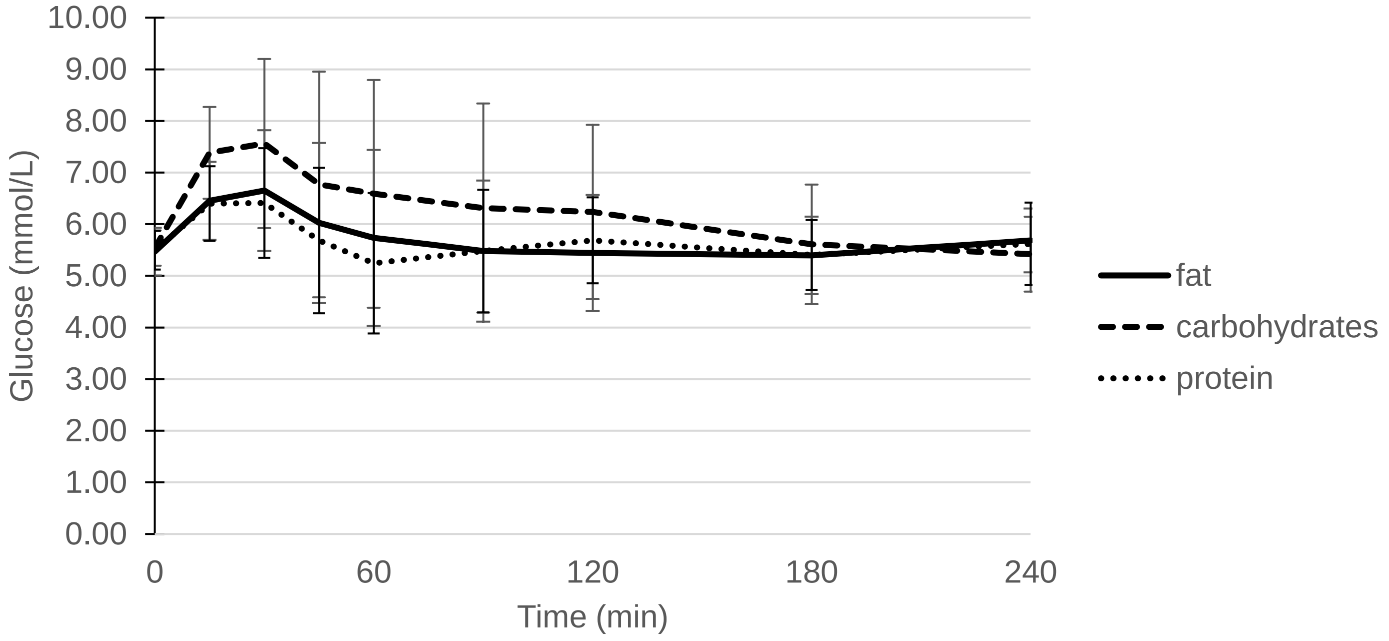


**B**

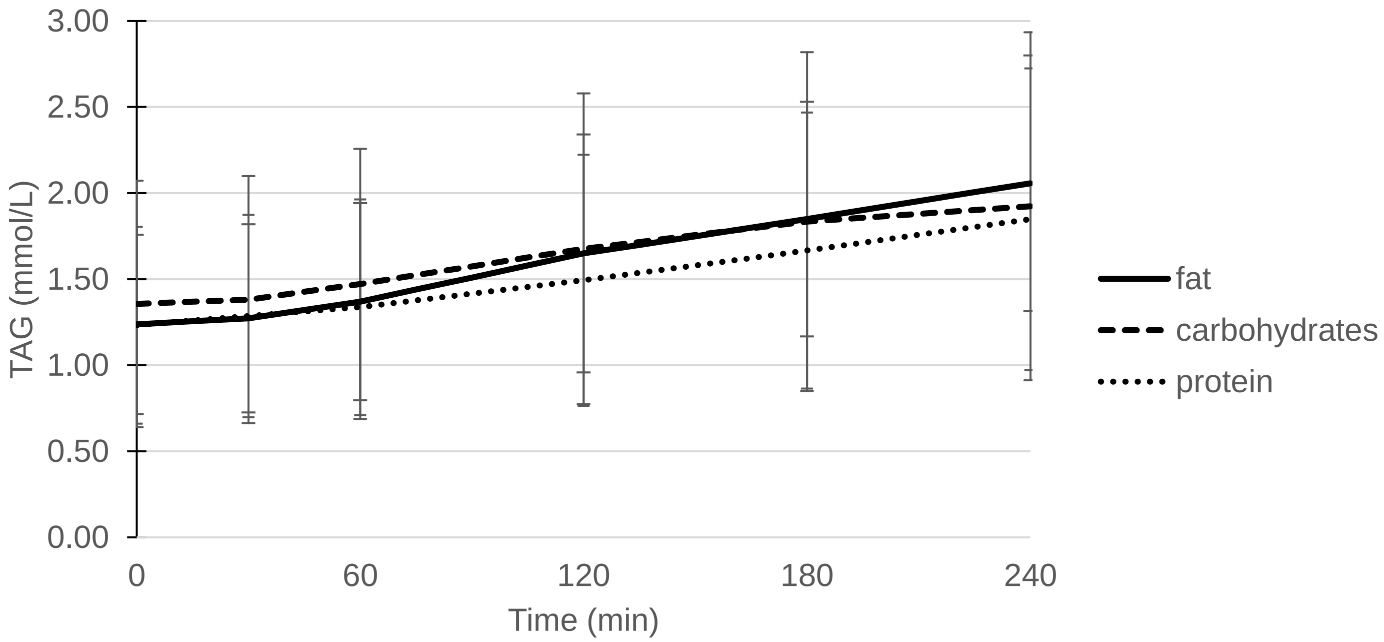

**C**

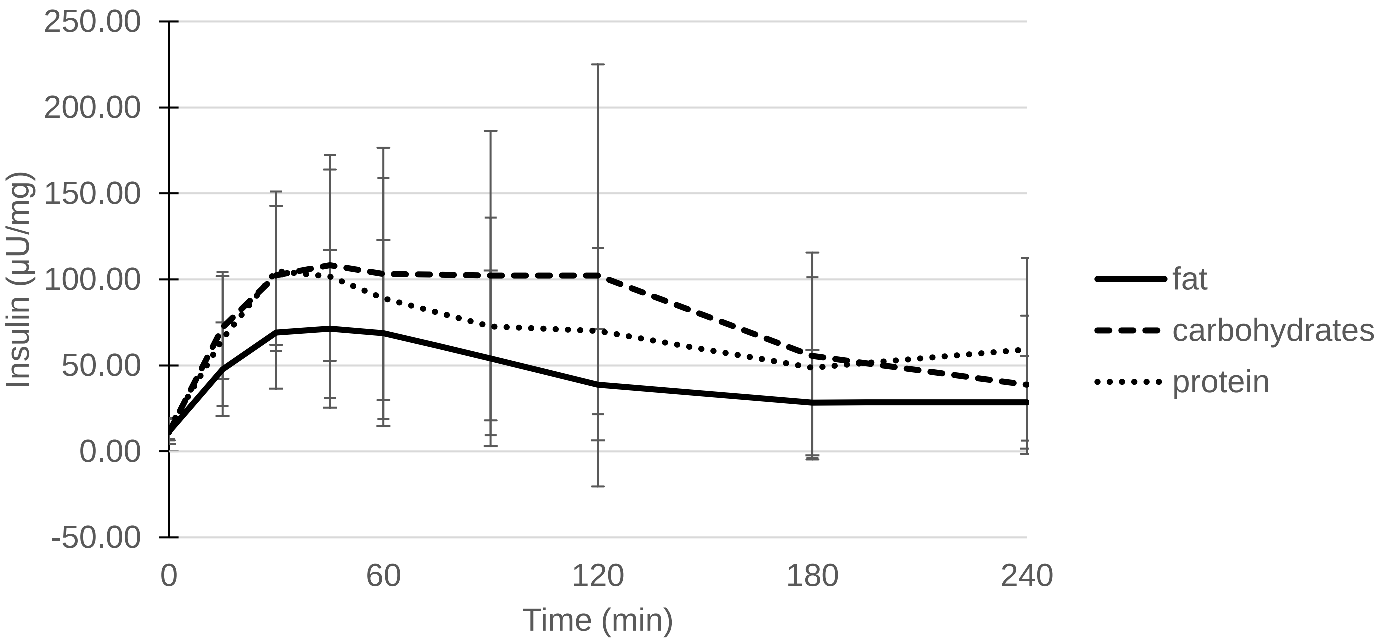


**D**

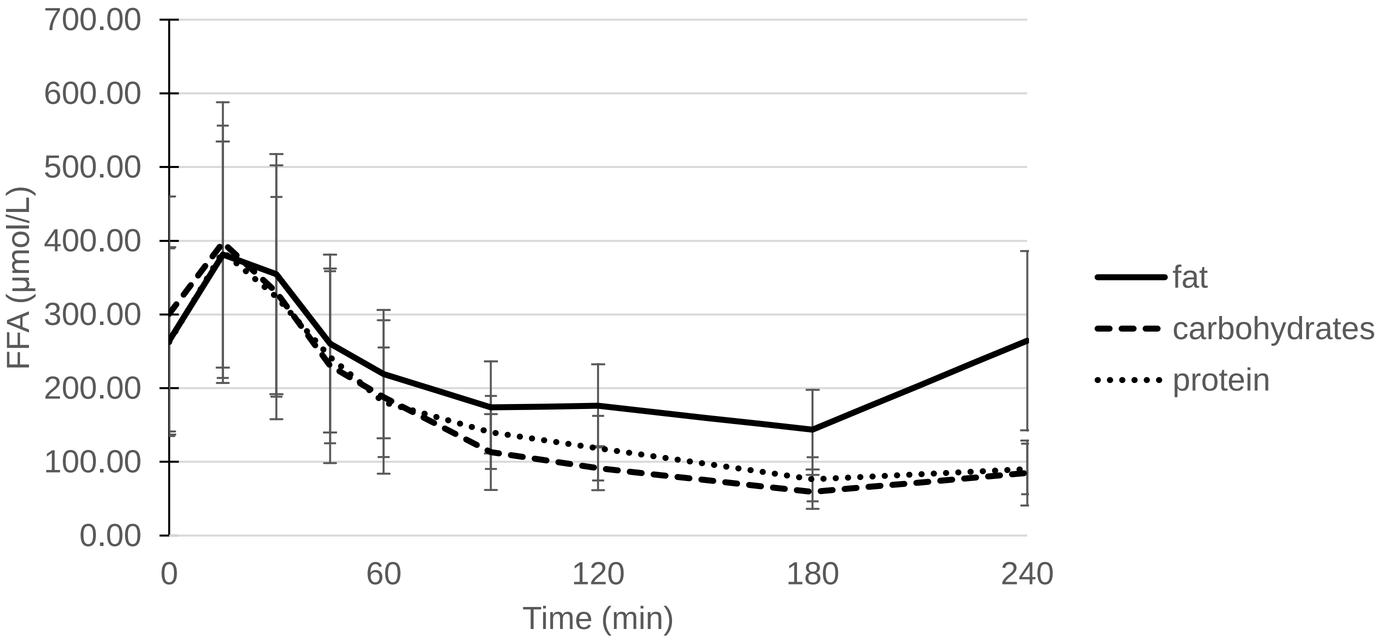


*TAG: triacylglycerol; FFA: free fatty acids*
